# Polymorphisms in the glucocorticoid receptor gene and in the glucocorticoid-induced transcript 1 gene are associated with disease activity and response to glucocorticoid bridging therapy in rheumatoid arthritis

**DOI:** 10.1007/s00296-015-3235-z

**Published:** 2015-02-28

**Authors:** R. A. M. Quax, J. W. Koper, A. M. Huisman, A. Weel, J. M. W. Hazes, S. W. J. Lamberts, R. A. Feelders

**Affiliations:** 1 Department of Internal Medicine, Erasmus MC, University Medical Center, P.O.B.2040, 3000 CA Rotterdam, The Netherlands; 2 Department of Rheumatology, Erasmus MC, University Medical Center, Rotterdam, The Netherlands; 3 Department of Rheumatology, Sint Franciscus Gasthuis, Kleiweg 500, 3045 PM Rotterdam, The Netherlands; 4 Department of Rheumatology, Maasstad Hospital, Maasstadweg 21, 3079 DZ Rotterdam, The Netherlands

**Keywords:** Rheumatoid arthritis, Glucocorticoid sensitivity, Glucocorticoid receptor gene polymorphisms, Glucocorticoid-induced transcript 1 gene polymorphisms

## Abstract

Glucocorticoids (GC) are widely used in rheumatoid arthritis (RA). Ongoing active disease due to GC resistance may unfavorably influence long-term disease outcome in RA. We studied the association between the presence of glucocorticoid receptor (*GR*) and glucocorticoid-induced transcript 1 (*GLCCI1*) gene polymorphisms, which modulate GC sensitivity, and baseline disease activity score (DAS) and efficacy of GC bridging therapy in RA. We prospectively studied in vivo GC sensitivity in 138 patients with recent-onset or longstanding RA. In vivo GC sensitivity was expressed as the relative decrease in DAS following 2 weeks of standardized GC therapy. All patients were genotyped for the GR polymorphisms *Bcl*I (rs41423247), N363S (rs6195), 9β (rs6198), ER22/23EK (rs6189 + rs6190), and the GLCCI1 variant rs37972 and subsequently divided in groups carrying a polymorphism associated with increased GC sensitivity (*Bcl*I-G allele, N363S-G allele, GLCCI1-C allele) or decreased GC sensitivity (9β-G allele, ER22/23EK-A/A allele, GLCCI1-T allele). Differences in baseline DAS and relative decrease in DAS in the different genotype groups were analyzed using analysis of covariance and linear regression. Baseline DAS was higher in patients who carried polymorphisms of the GR and GLCCI1 genes associated with decreased GC sensitivity. GLCCI1 genotype, but not GR genotypes, was associated with improvement in DAS in male patients with RA. The GLCCI1 gene minor allele (rs37972) may be associated with less efficient GC bridging therapy in male RA patients. Carriers of the *Bcl*I-G, N363S-G, or GLCCI1-C alleles had lower levels of baseline disease activity, suggesting a role for the GLCCI1 and GR gene in regulation of GC sensitivity to *endogenously* produced cortisol.

## Introduction

Rheumatoid arthritis (RA) is an autoimmune disease characterized by chronic inflammation of the synovial lining which ultimately, when left untreated, leads to erosive disease of the joints. To date, therapeutic strategies for the treatment of recent-onset RA are characterized by initiation of aggressive (combination) therapy in the ‘window of opportunity’, as this has been shown to improve long-term outcome of RA [1–4]. GCs are frequently prescribed as ‘bridging’ therapy, referring to the time period needed for newly initiated disease modifying antirheumatic drugs (DMARDs) to become clinically effective. Recently, we have demonstrated that a poor initial response to GC bridging therapy is a strong predictor for DMARD failure at 3-months follow-up [5]. These findings underscore the importance of the primary response to GC treatment and prompted us to further identify determinants of GC sensitivity. Individual GC sensitivity is highly variable and is determined by both genetic and acquired factors [6]. In this context, the recent findings of Tantisira and co-workers reveal that the functional mutant T allele of SNP rs37972, mapping to the glucocorticoid-induced transcript 1 gene (GLCCI1), is associated with declined efficacy of GC inhalation therapy in asthmatic patients are of special interest [7]. We hypothesized that GLCCI1-mediated differences in clinical response to GC may also apply to patients with RA (class effect in pharmacogenetics). In addition, four functionally well-characterized polymorphisms of the glucocorticoid receptor (GR) gene (9β, ER22/23EK, *Bcl*I and N363S) that modulate GC sensitivity could also partly explain variation in clinical response to GC in patients with RA, as has been shown in other inflammatory diseases [8–12].

Therefore, the aim of this study was to examine whether the presence of polymorphisms of the GR and GLCCI gene is associated with disease activity and the clinical effect of GC bridging therapy in recent-onset and longstanding RA.

## Patients and methods

### Patients

This study was embedded in a multicenter randomized clinical trial studying persons older than 18 years presenting with a recent-onset arthritis, the so-called tREACH study (***t***reatment in the ***R***otterdam ***e***arly ***a***rthritis co***h***ort). The outline of this study has been described in detail [13]. All tREACH patients with definite RA and disease activity scores before start and after 2 weeks of GC treatment were included in the current study (*N* = 112).

In an independent cohort, 26 patients with established RA and active disease were recruited (FLARE study). Active disease was defined as disease activity requiring GC therapy according to the treating rheumatologists [14].

All patients received standardized GC therapy, either 15 mg prednisone daily or a single intramuscular depot of methylprednisolone 120 mg or triamcinolone acetonide 80 mg. All tREACH patients were GC and DMARD naive. FLARE patients had not used GC for at least the last 3 months and were on stable DMARD therapy.

### Methods

#### In vivo glucocorticoid sensitivity

Trained research nurses examined patients before and after 2 weeks of standardized GC treatment using a standardized 44-joint count for swelling and pain. Disease activity was objectively scored using the DAS44 with four variables [swollen joint count (SJC), Ritchie Articular Index (RAI), erythrocyte sedimentation rate (ESR), and general health at a 100 mm scale (GH)], hereafter referred to as DAS [15]. The relative decrease in DAS (%) [(DAS_baseline_ − DAS_after 2 weeks_)/DAS_baseline_) × 100] was used as an index for in vivo GC sensitivity. Relative improvement of the individual measures of the DAS was calculated similarly. Use of non-steroidal anti-inflammatory drugs (NSAIDs) and, if applicable, use of DMARDs and tumor necrosis factor-alpha (TNF-α) blocking agents was recorded at baseline.

#### Glucocorticoid receptor polymorphisms

All 138 patients were genotyped for four functional polymorphisms of the GR gene (ER22/23EK, rs6189 and rs6190; N363S, rs6195; *Bcl*I, rs41423247 and 9β, rs6198) and the GLCCI1 gene variant (rs37972). DNA was extracted from samples of peripheral venous blood samples using standard techniques. Genotyping was performed using Taqman allelic discrimination assays (Applied Biosystems), following protocols described by the supplier. Genotyping of *Bcl*I failed in one patient, in two patients each the N363S, and GLCCI1 genotype could not be determined. Results were analyzed using the sequence detection system 2.2 software (Applied Biosystems).

#### Statistical analysis

Differences between groups of the baseline characteristics were tested using ANOVA or Mann–Whitney *U* test for continuous variables and Pearson’s chi-square test for categorical variables.

**Fig. 1:**
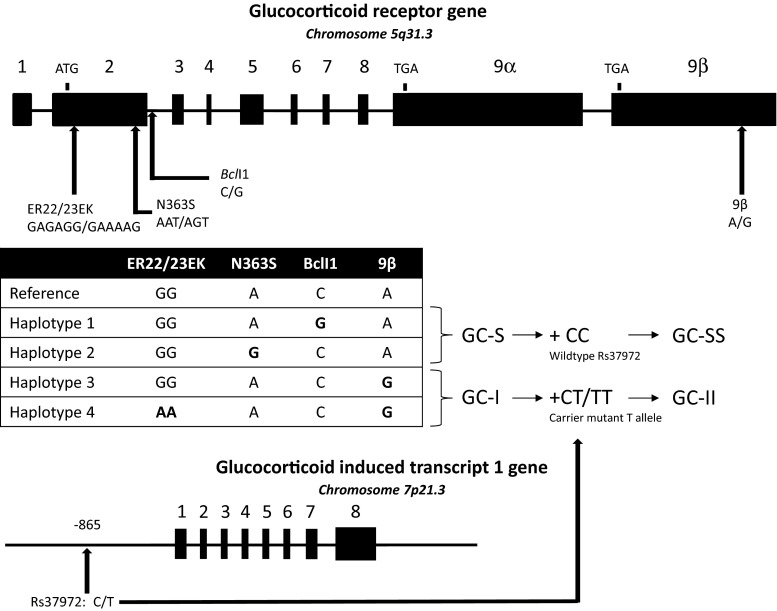
Polymorphisms in the GR and GLCCI1 genes. The GR gene consists of nine exons and is located on chromosome 5. The *Bcl*I (mutant G allele) and N363S (mutant G allele) SNPs have been associated with increased GC sensitivity in vivo, whereas the 9β (mutant G allele) and the ER22/23EK (GA**G**A**G**G → GA**A**A**A**G) polymorphisms have been linked to decreased GC sensitivity in vivo. Rs37972 is located 865 base pairs upstream of exon 1 of the GLCCI1 gene. The GC-S group consists of all patients with GR haplotype 1 and/or haplotype 2 and did not carry haplotype 3 or 4. Patients in the GC-S group who also carried the wild-type GLCCI1 genotype were classified as GC-SS. Patients with GR haplotype 3 or 4, and did not carry haplotype 1 or 2, were grouped as GC-I. Patients in the GC-I group who also carried one or two mutant GLCCI1-T alleles were attributed to the GC-II group

The different GR haplotypes were classified based on their known functional associations with GC sensitivity (Fig. 1). Patients with haplotype 1 (*Bcl*I, mutant G allele) and 2 (N363S, mutant G allele) were merged and referred to as the GC-S (increased GC sensitivity) group (*N* = 72). Patients with haplotype 3 (9β, mutant G allele) and 4 (ER22/23EK, mutant alleles A and A + 9β, mutant G allele) were combined and referred to as the GC-I (decreased GC sensitivity) group (*N* = 29). Patients who carried both haplotype 1/2 and 3/4 were excluded.

In addition, a new genotype variable was composed, combining the allele variants of the GR and GLCCI1 genes associated with increased and decreased GC sensitivity, respectively, (GC-SS: GC-S group + homozygous carriers of the GLCCI1-C wild-type allele, *N* = 30; GC-II: GC-I group + carriers of one or two minor GLCCI1-T alleles, *N* = 19).

We used analysis of covariance to analyze baseline DAS in carriers and non-carriers of the GR and the GLCCI1 gene polymorphisms and adjusted for age, gender, and use of NSAIDs. Relative decrease in DAS in the different genotype groups was analyzed using linear regression (relative decrease in DAS as dependent variable; use of NSAIDs as covariate). Male and female patients were analyzed separately (significant interaction gender–GLCCI1 genotype).

Differences between individual measures of the DAS (SJC, RAI, ESR, and GH) in the different genotype groups were analyzed using the Kruskal–Wallis test with post hoc Dunn’s correction for multiple comparisons.

All analyses were performed using the statistical package of SPSS for windows, version 17.0 (Chicago, IL, USA) and GraphPad Prism, version 5.01 (La Jolla, CA, USA). We considered differences statistically significant if *p* ≤ 0.05 (two-sided).

#### Ethical approval

All subjects signed informed consent, and the study was approved by the medical ethics committee of the Erasmus Medical Center.

## Results

A total of 138 patients were included in the current study of whom 73 patients were treated with oral GC and 65 patients were given an intramuscular depot of GC. Further baseline characteristics of the study population are shown in Table 1. The frequencies of the individual GR polymorphisms (Table 2) in our cohort were similar to those we observed in earlier, larger cohorts [16].

**Table 1:** Patients characteristics

	Early RA	Early RA	Established RA
	Oral GC bridging (*N* = 73)	Intramuscular GC bridging (*N* = 39)	Intramuscular GC bridging (*N* = 26)
Female gender, *N* (%)	43 (58.9)	26 (66.7)	16 (61.5)
Age, mean (SD)	54.5 (12.6)	53.3 (15.3)	55.7 (12.1)
Body mass index, median (range)	25.6 (17.5–43.1)	25.5 (20.3–39.2)	25.9 (18.9–40.4)
Disease duration (months), median (range)	5.7 (1.2–11.9)^a^	4.6 (0.5–11.9)^a^	67.00 (0–282)
Rheumatoid factor (IgM) positive, *N* (%)	59 (80.8)	32 (82.1)	20 (76.9)
Anti-CCP positive, *N* (%)	64 (87.7)	31 (79.5)	18 (90.0)^c^
Presence of joint erosions, *N* (%)	5 (6.8)^a,b^	10 (25.6)^a^	14 (53.8)
DAS44 at baseline, mean (SD)	3.21 (0.99)	3.25 (0.74)	3.33 (0.98)
Use of NSAID, *N* (%)	52 (71.2)	27 (69.2)	15 (57.7)
Use of methotrexate, *N* (%)	–	–	15 (57.7)
Use of hydroxychloroquine, *N* (%)	–	–	7 (26.9)
Use of sulfasalazine, *N* (%)	–	–	4 (15.4)
Number of DMARDs, median (range)	–	–	1 (0–3)^d^
Use of anti-TNF therapy, *N* (%)	–	–	3 (11.5)

DAS44: Disease activity score, 44 joints; anti-CCP: anti-cyclic citrullinated protein

^a^ *p* < 0.05 compared to established RA, intramuscular GC bridging

^b^ *p* < 0.05 compared to early RA, intramuscular GC bridging

^c^Anti-CCP was not routinely analyzed, % is based on 20 patients with known anti-CCP status

^d^7 patients were not using any DMARD at time of assessment

**Table 2:** Frequencies of individual polymorphisms of the GR and GLCCI1 gene

Polymorphisms		Nucleotide change	Wildtype	Heterozygous carrier	Homozygous carrier
*Bcl*I	*rs41423247*	C→G	52 (38)	70 (51.1)	15 (10.9)
9β	*rs6198*	A→G	89 (64.5)	42 (30.4)	7 (5.1)
ER22/23EK	*rs6189* and* rs6190*	G→A and G→A	129 (93.5)	8 (5.8)	1 (0.7)
N363S	*rs6195*	A→G	128 (94.1)	8 (5.9)	0 (0)
GLCCI1	*rs37973*	C→T	53 (39)	63 (46.3)	20 (14.7)

Genotyping of *Bcl*I failed in one patient; in two patients each, the N363S and GLCCI1 genotype could not be determined. The *Bcl*I polymorphism is located in an intron. The 9β SNP is located in an ATTTA motif (ATTTA → GTTTA) and is associated with increased stability of the GR-β mRNA and a reduced transrepressive capacity in vitro. The nucleotide change in the ER22/23EK variant results in a different amino acid (glutamic acid–arginine → glutamic acid–lysine) and has been related to increased expression of the transcriptionally less active GR-A isoform. Similarly, the A to G nucleotide change in the N363S polymorphism alters asparagine into serine. In vitro assays demonstrated reduced luciferase reporter activity in cells transfected with a rs37973 reporter construct. Data are presented as *N* (%)

### Glucocorticoid receptor and GLCCI1 gene polymorphisms and disease activity

**Fig. 2:**
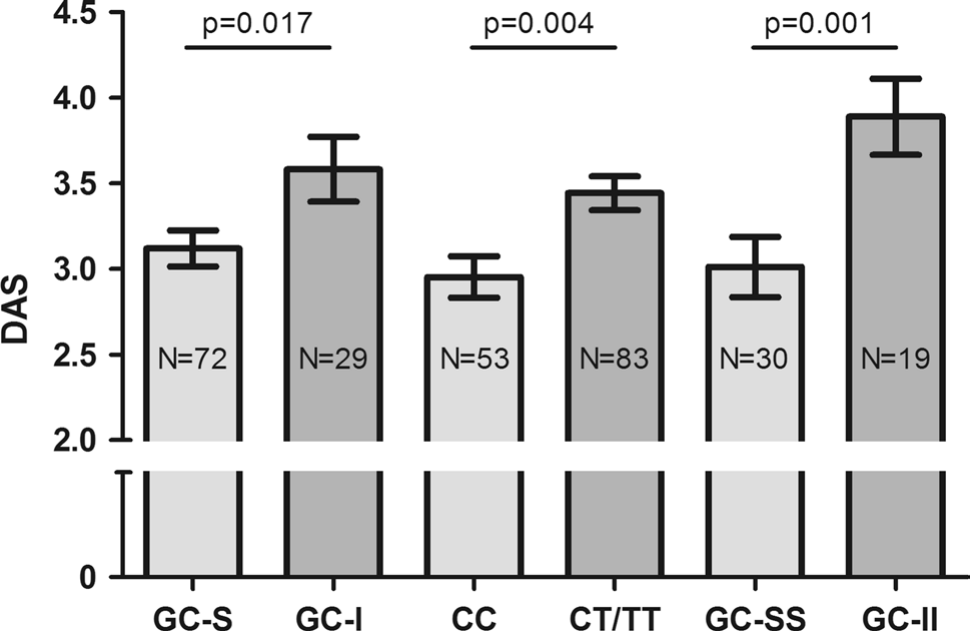
Baseline DAS in relation to GR and GLCCI1 gene polymorphisms. *Baseline* DAS in GC-S versus GC-I, patients with wild-type GLCCI1-C alleles versus carriers of the GLCCI1-T variant and patients with both the GC-S and GLCCI1-C variant (GC-SS) versus patients with both the GC-I and GLCCI1-T variant (GC-II); adjusted for age, gender, and use of NSAIDs

The majority of patients had moderately to highly active RA (DAS ≥ 2.4, *N* = 117, 84.8 %). Patients in the GC-I (haplotype 3 or 4) and GC-II (GC-I and carrier of one or two minor GLCCI1-T alleles) groups and carriers of the GLCCI1 mutant T allele all had significantly higher baseline disease activity than their respective reference genotype group or wild-type allele, as expected based upon the functional associations of these genotype variants (Fig. 2). Use of NSAIDs was also independently associated with higher baseline DAS (*p* < 0.05; data not shown).

### Glucocorticoid receptor and GLCCI1 gene polymorphisms and response to GC bridging therapy

**Fig. 3:**
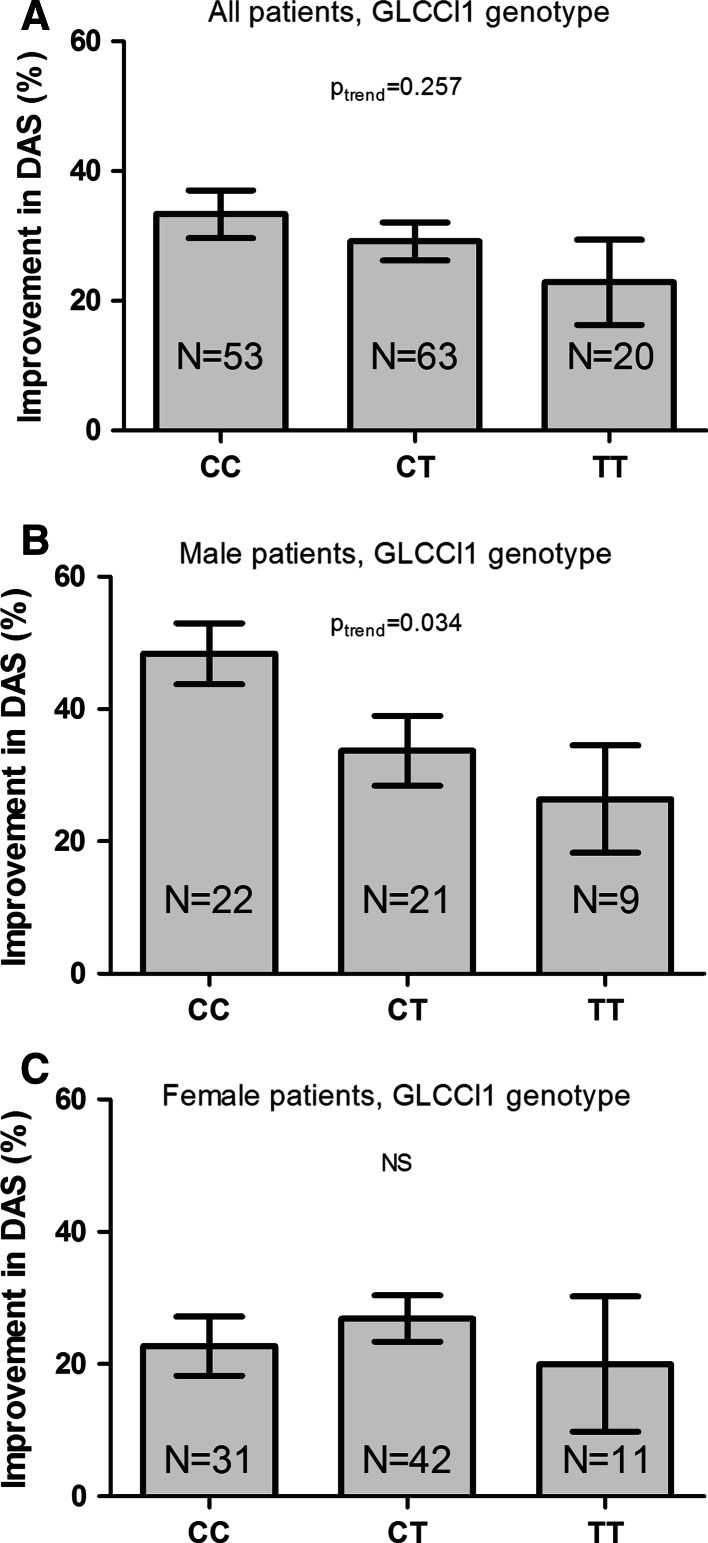
The relative improvement in DAS following GC bridging therapy in relation to GLCCI1 gene polymorphisms. Percentual improvement in DAS after 2 weeks of standardized GC treatment in all patients (**a**) male patients (**b**) and female patients (**c**). *Error bars* represent standard error of the mean (SEM); adjusted for use of NSAIDs

After 2 weeks of GC bridging therapy, the mean (SD) relative decrease in DAS in all patients was 30.2 (25.4) %. Orally and intramuscularly treated patients displayed similar responses (oral GC: 32.0 (21.7) % and intramuscular GC: 28.3 (29.0) %). Male and female patients had significantly different levels of relative decrease in DAS (men 38.6 (24.1) %; women 25.1 (24.9) %, *p* = 0.002). When all patients were analyzed together, no trend between GLCCI1 genotype and relative decrease in DAS was observed (Fig. 3a, *p*_trend_ = 0.257).

Within men, however, carriers of the mutant GLCCI1-T allele had relatively less improvement in DAS as compared to patients who only have the wild-type C allele (Fig. 3b, *p*_trend_ = 0.034). A comparable pattern was observed when male patients in the GC-SS and GC-II groups were studied although this did not reach statistical significance (*p* = 0.056), probably due to small numbers of patients (data not shown).

**Fig. 4:**
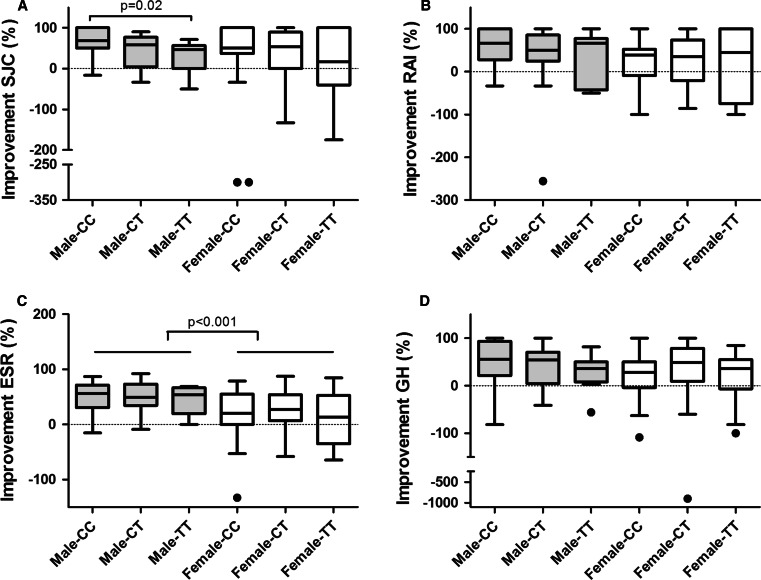
Relative improvement of SJC (**a**) RAI (**b**) ESR (**c**) and GH (**d**) in male and female patients with RA after 2 weeks of GC bridging therapy, according to GLCCI1 genotype (Tukey box-and-Whisker plot). Relative improvement of each individual measure of the DAS was calculated as 100 × [(baseline value-2 weeks value)/baseline value]. In eight patients, the relative improvement in RAI could not be calculated because the baseline value was zero (two male CC, two female CC, two male CT and, two female CT patients). Negative values indicate higher levels of SJC/RAI/ESR/GH after 2 weeks of GC bridging therapy as compared to their baseline values. SJC = swollen joint count, RAI = Ritchie Articular Index, ESR = erythrocyte sedimentation rate, GH = general health at a 100 mm scale. Please note the different scales on the Y-axis

**Fig. 5:**
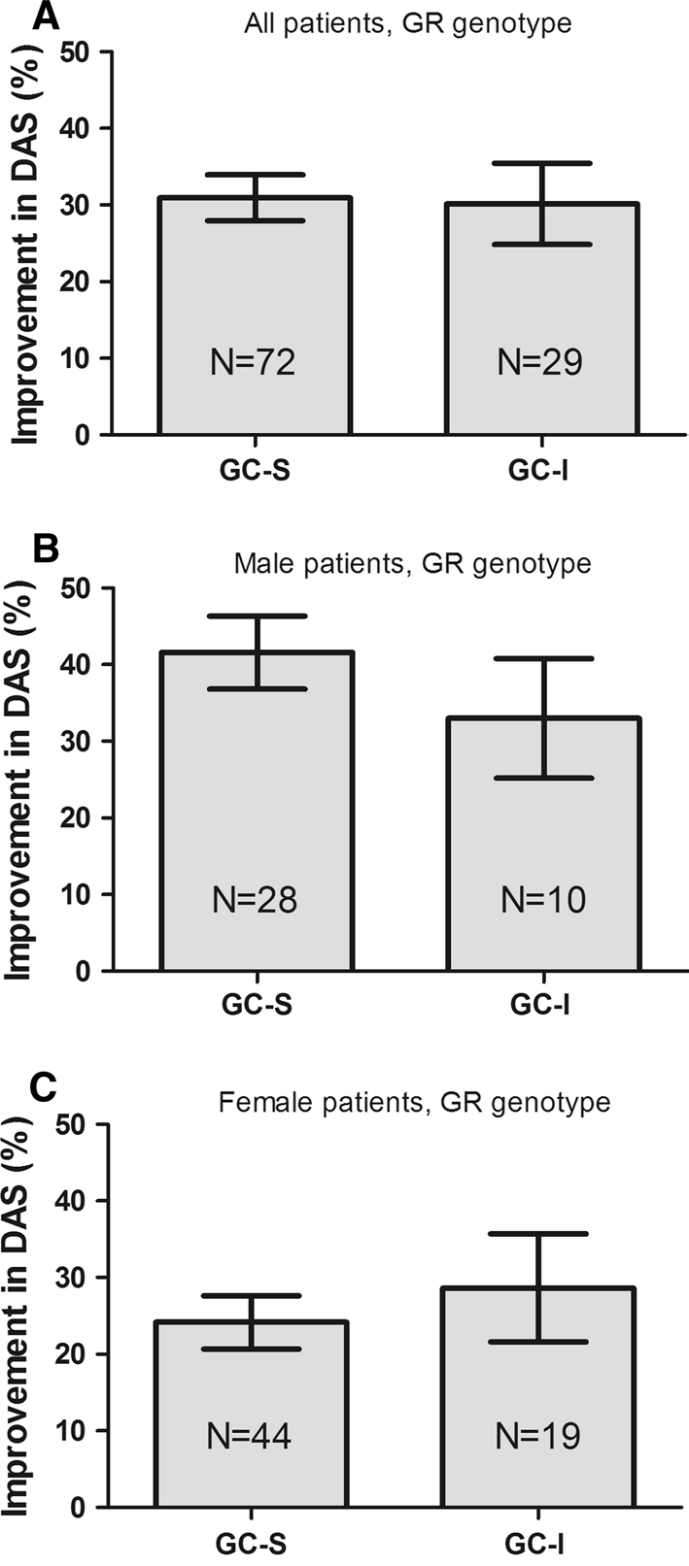
Relative improvement in DAS following GC bridging therapy in relation to GR gene polymorphisms. There were no differences in improvement in DAS when patients were stratified according to the GC-S versus GC-I model (**a**). Male patients (**b**), in general had better responses than female patients (**c**) following GC bridging therapy, but within group analyses of men and women did not show differences in relative decrease in DAS according to GR genotype. Patients who were homozygous carrier of the wild-type allele for all four studied GR polymorphisms are not depicted (*N* = 17). The *bars* represent the mean and standard error of the mean

This sexual dimorphic effect of improvement in DAS according to GLCCI1 genotype was further explored by studying the individual measures of the DAS (SJC, RAI, ESR, and GH), as visualized in Fig. 4. The relative improvement in SJC was significantly associated with GLCCI1 genotype in male patients (Fig. 4a), whereas relative improvement in RAI, ESR, and GH was not associated with GLCCI1 genotype (Fig. 4b–d). Improvement in DAS and/or SJC was not associated with GLCCI1 genotype in women (Figs. 3c, 4a). Apart from GLCCI1 genotype, relative improvement in ESR was significantly higher in male than in female patients (Fig. 4c). Absolute levels of SJC, RAI, ESR, and GH at baseline and after 2 weeks of GC bridging therapy are provided in Table 3. Patients with GR haplotype 1 or 2 (GC-S) and patients with GR haplotype 3 or 4 (GC-I) did not differ significantly in their level of improvement in DAS after 2 weeks of GC treatment. A subsequent subanalysis of men and women separately also did not reveal any difference in improvement in DAS according to GR genotype status (Fig. 5).

**Table 3:** DAS and individual measures of the DAS

	Men			Women		
	CC (*N* = 22)	CT (*N* = 21)	TT (*N* = 9)	CC (*N* = 31)	CT (*N* = 42)	TT (*N* = 11)
DAS44 at baseline, mean (SD)	2.95 (0.97)	3.62 (1.13)	3.19 (0.73)	2.96 (0.83)	3.42 (0.82)	3.39 (0.92)
SJC-baseline	7.5 (2–23)	12 (2–25)	8 (2–21)	5 (1–19)	7.5 (1–23)	5 (2–14)
RAI-baseline	5 (0–19)	6 (0–20)	5 (2–12)	7 (0–15)	7 (0–31)	7 (2–50)
ESR-baseline	21(1–74)	26.5 (9–76)	16 (3–81)	18 (1–51)	25.5 (5–69)	24 (13–78)
GH-baseline	45.5 (9–100)	50.0 (5–95)	47 (11–80)	52 (9–91)	59.5 (5–99)	50 (1–83)
DAS44-2 weeks, mean (SD)	1.53 (0.74)	2.47 (1.33)	2.40 (1.02)	2.24 (0.90)	2.50 (0.99)	2.76 (1.29)
SJC-2 weeks	2.5 (0–7)	5 (2–26)	5 (2–11)	2 (0–12)	3.5 (0–14)	5 (0–12)
RAI-2 weeks	1 (0–8)	4 (0–32)	3 (0–16)	4 (0–18)	5 (0–25)	7 (0–19)
ESR-2 weeks	8 (1–55)	13 (1–71)	11 (1–47)	13 (1–42)	15 (2–69)	21 (6–28)
GH-2 weeks	15 (0–69)	23 (0–100)	40 (2–67)	30 (0–76)	32 (0–100)	30 (2–80)

Absolute levels of DAS and individual measures of the DAS at baseline and after GC bridging therapy according to GLCCI1 genotype and gender. Individual measures of the DAS are given as median (range)

*SJC* swollen joint count, *RAI* Ritchie articular index, *ESR* erythrocyte sedimentation rate, *GH* general health at a 100 mm scale

## Discussion

GCs are widely used in RA and as in other inflammatory disorders; GC resistance is a well-known phenomenon which may lead to ongoing active disease [6, 17]. Accumulating evidence supports the existence of a ‘window of opportunity’ and recent guidelines for treating RA, therefore emphasizing the importance of reaching low disease activity as soon as possible [18, 19]. In this context, prediction of efficacy of GC bridging therapy is of great value, in particular since we have shown that a poor GC response after 2 weeks is a strong predictor of DMARD failure at 3 months [5]. We show for the first time that the GLCCI1 gene minor allele (rs37972) is associated with higher RA disease activity and, in male RA patients, with a lower clinical response to GC bridging therapy.

Individual GC sensitivity is influenced by genetic factors, cellular GR number, and affinity and GC availability [6, 17]. GR polymorphisms have been associated with differences in metabolic parameters and body composition, susceptibility to and severity of inflammatory diseases, and response to GC therapy [8, 9]. In RA, polymorphisms of the GR gene associated with reduced (i.e., 9β) or increased (i.e., *Bcl*I and N363S) GC sensitivity are associated with increased and decreased susceptibility, respectively, to RA. Moreover, patients using TNF-α blocking agents more frequently carried the ER22/23EK variant [16]. Increased expression of the transcriptionally less active GR-A (translational) isoform due to the ER22/23EK polymorphism might be one of the underlying mechanisms of GC resistance [10]. GC resistance in association with the 9β polymorphism might be related to increased stability of the GRβ mRNA (which in turn may lead to higher levels of the dominant negative GRβ protein) and decreased GC-related transrepression of pro-inflammatory genes [11, 12]. Tantisara and co-workers recently identified variations in the GLCCI1 gene as a factor associated with responsiveness to inhaled GC in asthmatic patients, and van den Berge et al. [7, 20] could reproduce these findings in patients with COPD.

Although little is known with regard to the function of GLCCI1, dexamethasone-induced apoptosis of immune cells might be one of the mechanisms influenced by rs37972 [7, 21]. As GC-mediated apoptosis is an important mechanism via which GC modulate inflammatory processes, we hypothesized that the GLCCI1 genotype might also influence the clinical response to exogenously administered GC in RA. In our prospectively studied cohort, we found a gender-specific association of GLCCI1 genotype and response to GC bridging therapy. Several studies in RA report a more severe disease course in women with less beneficial effects of (combination) DMARD therapy and of biologic agents [22–24]. The worldwide QUEST-RA study suggests that these differences might be based on a different composition of the DAS by its individual measures in male and female patients (i.e., higher DAS in women at similar numbers of swollen joints) [25]. In our study, however, also the relative improvement in SJC, as the most objective individual measure of the DAS, was not associated with GLCCI1 genotype in female patients. This suggests a true gender-specific effect in men, although the exact mechanism remains to be elucidated. Of note, gene–gender interactions are known in a variety of clinical disorders [26].

Furthermore, carriers of minor alleles associated with decreased GC sensitivity (ER22/23EK, 9β, and GLCCI1) had higher DAS at baseline. This implies that GR and GLCCI1 gene polymorphisms might also be involved in modulation of effects of *endogenously* produced cortisol, herewith influencing the immunosuppressing effects of cortisol. This might be true in particular for RA, since RA is characterized by relatively low cortisol levels, which are not in proportion with the degree of inflammation in these patients. This increases the importance of GC sensitivity for *endogenously* produced cortisol [27]. We cannot fully exclude the possibility that differences in endogenous cortisol levels influenced the disease activity scores at baseline, although we did not find a correlation between basal (08AM) salivary cortisol levels and DAS in a subset of our patients (*N* = 49) in whom basal salivary cortisol was measured as part of another substudy (data not shown).

Although the exploring character of this study underscores the potential of GLCCI1 genotyping as marker of GC sensitivity, relatively small numbers of patients have been included. Clearly, our findings require validation in other (larger) cohorts with (early) RA but might also incite to explore rs37972 functionality in other inflammatory diseases frequently treated with GC (e.g., polymyalgia rheumatica, inflammatory bowel disease). Furthermore, RA is considered to be a polygenic disease, and other polymorphisms are likely to be related to disease activity and/or response to GC treatment as has been shown for instance for FKBP5 in Crohn’s disease [28]. In addition to genetic variation, many other factors are likely to influence GC sensitivity and the response to endogenous cortisol and exogenously administered GC (e.g., metabolism of GC, inflammatory status) [6].

In conclusion, we show that GLCCI1 gene variants might be associated with response to GC bridging therapy in male patients with RA. In addition, GR and GLCCI1 gene polymorphisms may be associated with baseline disease activity in RA, suggesting that differences in the response to *endogenously* produced GC are also partly mediated via GR and GLCCI1 gene polymorphisms.

## Abbreviations

GC: Glucocorticoids
RA: Rheumatoid arthritis
GR: Glucocorticoid receptor
GLCCI1: Glucocorticoid-induced transcript 1
DAS: Disease activity score
DMARDs: Disease modifying antirheumatic drugs
SJC: Swollen joint count
RAI: Ritchie Articular Index
ESR: Erythrocyte sedimentation rate
GH: General health at a 100 mm scale
NSAIDs: Non-steroidal anti-inflammatory drugs;
TNF-α: Tumor necrosis factor-alpha
GC-S group: Carriers of BclI (haplotype 1) and/or N363S (haplotype 2)
GC-I group: Carriers of 9β (haplotype 3) or 9β + ER22/23EK (haplotype 4)
GC-SS: GC-S group + homozygous carriers of the GLCCI1-C wild-type allele
GC-II: GC-I group + carriers of one or two minor GLCCI1-T alleles
